# Robust Genetic Transformation System to Obtain Non-chimeric Transgenic Chickpea

**DOI:** 10.3389/fpls.2019.00524

**Published:** 2019-04-26

**Authors:** Sudipta Shekhar Das Bhowmik, Alam Yen Cheng, Hao Long, Grace Zi Hao Tan, Thi My Linh Hoang, Mohammad Reza Karbaschi, Brett Williams, Thomas Joseph V. Higgins, Sagadevan G. Mundree

**Affiliations:** ^1^Centre for Tropical Crops and Biocommodities, Queensland University of Technology, Brisbane, QLD, Australia; ^2^CSIRO Agriculture and Food, Canberra, ACT, Australia

**Keywords:** *Agrobacterium*, transgenic chickpea, chimeric chickpea, micro-injury of *in vitro* explants, LED light, legume transformation

## Abstract

Chickpea transformation is an important component for the genetic improvement of this crop, achieved through modern biotechnological approaches. However, recalcitrant tissue cultures and occasional chimerism, encountered during transformation, hinder the efficient generation of transgenic chickpeas. Two key parameters, namely micro-injury and light emitting diode (LED)-based lighting were used to increase transformation efficiency. Early PCR confirmation of positive *in vitro* transgenic shoots, together with efficient grafting and an extended acclimatization procedure contributed to the rapid generation of transgenic plants. High intensity LED light facilitate chickpea plants to complete their life cycle within 9 weeks thus enabling up to two generations of stable transgenic chickpea lines within 8 months. The method was validated with several genes from different sources, either as single or multi-gene cassettes. Stable transgenic chickpea lines containing GUS (*uidA*), stress tolerance (*AtBAG4* and *TlBAG*), as well as Fe-biofortification (*OsNAS2* and *CaNAS2*) genes have successfully been produced.

## Introduction

Chickpeas are an important grain legume with an annual global production of 14.2 million tons in 2014 (FAOSTAT, 2016). The demand for high quality chickpeas is rising steadily with the increasing needs of the Indian subcontinent and is predicted to reach 17.0 Mt by 2020 (Abate and Orr, 2012). Global chickpea productivity does not meet current demand because of several abiotic and biotic constraints. Furthermore, the area under chickpea cultivation has been stagnant (Thudi et al., 2014). Although conventional breeding and enhanced management practices have addressed several chickpea production constraints as well as improved grain quality, the lack of diverse germplasms for certain traits, sexual incompatibility and a high degree of autogamy continue to limit chickpea yields (Van Rheenen et al., 1993; Somers et al., 2003; Atif et al., 2013). Genetic engineering (GE) is a powerful tool that has been used to improve the agronomic traits of other crops such as corn, cotton and soybean. GE involves crop improvement through the transfer of new genes for specific traits or by reducing the expression of selected genes using recombinant DNA technology. Recent advances in gene editing allow the precise manipulation of a crop’s genome without the integration of foreign DNA. To apply these powerful tools for crop improvement, an efficient regeneration and transformation system is a priority. Unfortunately, for most crops, transformation and regeneration remain difficult even after more than 30 years of technological advances (Altpeter et al., 2016).

Chickpeas are legumes with a limited genetic variation (Varshney et al., 2013; Roorkiwal et al., 2014) with minimal collections of its wild relatives such as *Cicer reticulatum* Ladiz and *Cicer echinospermum* P.H. Davis (Berger et al., 2003). Therefore, GE offers a promising tool for genetic improvement through the transfer of foreign genes from distantly related species to address pressing needs, such as increased resilience to drought, heat, cold, increased seed nutrient density, reduced dependence on inputs, and resistance to biotic stress. The foremost essential requirement for GE improvement requires efficient transformation methods which in chickpea have been considered challenging due to their recalcitrant responses to *in vitro* regeneration and genetic transformation (Chandra and Pental, 2003; Somers et al., 2003; Polowick et al., 2004; Varshney et al., 2009). In addition, chimerism (a single plant tissue containing transformed and non-transformed sections) in legumes is fairly common and a prime factor for non-transmission of genes to subsequent generations. For example, previous studies have reported approximately 22 and 29% non-transmitting, chimeric lines in chickpea and lentil, respectively (Christou, 1990; Dillen et al., 1997; Sarmah et al., 2004; Celikkol Akcay et al., 2009). Similarly, around 9% of total transgenic events in cowpea were also reported to be chimeric (Popelka et al., 2004). The presence of chimeric tissues reduces the efficiency of recovering stable transgenic lines (Christou, 1990; Dillen et al., 1997; Sarmah et al., 2004; Celikkol Akcay et al., 2009). Although several labs have reported chickpea transformation, limitations associated with the reproducibility of the technique (Huda et al., 2000), poor *in vitro* rooting (Polowick et al., 2004; Senthil et al., 2004), low transformation efficiency (Popelka et al., 2004; Sanyal et al., 2005; Mehrotra et al., 2011) and non-transmission of genes to subsequent generations (Krishnamurthy et al., 2000; Sarmah et al., 2004) remain problematic. Excluding a single report in lentil (Celikkol Akcay et al., 2009) which demonstrated reduced chimerism and stable expression of a GUS reporter in subsequent generations, measures for eliminating chimerism in other legumes including chickpea have not been reported. Here, we report the development of an efficient and reliable chickpea transformation system using improvements in current techniques that also reduce chimerism. This method is based on the *Agrobacterium*-mediated transformation of chickpea half-embryos using LED lighting and needle micro-injury as critical parameters. We demonstrate stable chickpea transgenics with genes related to stress tolerance and biofortification.

## Materials and Methods

### Experimental Design

**FIGURE 1:**
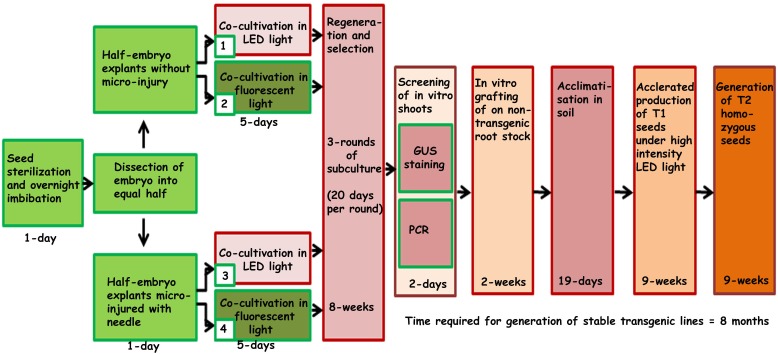
Schematic representation of steps involved in developing the improved protocol for *Agrobacterium*-mediated genetic transformation and regeneration of chickpea using half embryo explants.

Regeneration of chickpeas under *in vitro* conditions has previously been optimized (Sarmah et al., 2004). However, a further improvement in transformation efficiency and regeneration of stable transgenic chickpeas has proved a challenge. Half embryos (one cotyledon plus axis) were found to be the most promising explants to produce stable transgenic chickpeas (Sarmah et al., 2004). The current study aims to further increase the transformation efficiency by reducing chimerism, which is a major bottleneck for gene transmission to subsequent generations. To achieve this, we modified several important steps of the existing protocol. These included the concentration of kanamycin, type of light, a method of explants preparation including co-cultivation with *Agrobacterium*, duration of subculturing, acclimatization of T_0_ plantlets and early PCR screening. These changes are outlined in the protocol flow diagram (Figure 1).

### Vector Construction and Gene Constructs

**FIGURE 2:**
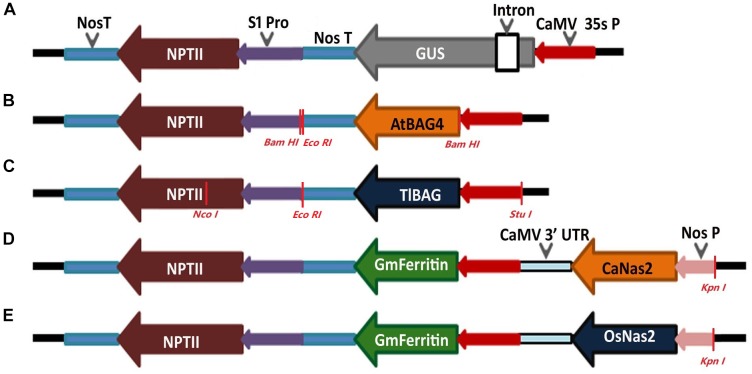
Schematic representation of the constructs made for chickpea plant transformation. **(A)** Reporter gene construct with constitutive promoter CaMV 35s driving *GUS* and *NPTII* gene; **(B,C)** Abiotic stress tolerant gene constructs with *AtBAG4* gene from Arabidopsis and *TlBAG* gene from *Tripogon loliiformis*, respectively, driven by CaMV 35s promoter and *NPTII* driven by S1 promoter for both genes; **(D,E)** Multigene construct for iron biofortification with chickpea *Nas2* (*CaNas2*) and rice *Nas2* (*OsNas2*) genes driven by Nos promoter, soybean *Ferritin* (*GmFerritin*) driven by CaMV 35s promoter and *NPTII* driven by S1 promoter.

To demonstrate the robustness of the protocol and stability of gene transmission in subsequent generations, chickpeas were transformed with five different gene constructs based on the binary vector pOPT-EBX (Figure 2). The binary vector pOPT-EBX was modified from pCAMBIA 2300 in Centre for Tropical Crops and Biocommodities, Queensland University of Technology, Queensland, Australia. These modifications included replacing the NOS promoter with the S1 promoter from subterranean clover stunt virus DNA segment (Schünmann et al., 2003) to drive the neomycin phosphotransferase II gene (NPTII) and inserting the CaMV35S promoter between Stu I and Sma I sites to drive genes of interest. Details of all the binary vectors used here and their transformation into *Agrobacterium tumefaciens* strain AGL1 were described previously (Tan et al., 2018). The genes in the different T-DNAs are shown schematically in Figure 2. The GUS (*uidA*) gene is a reporter gene that encodes the beta-glucuronidase enzyme. The BAG (Bcl-2 associated athanogene) functions as adapter proteins forming complexes with signaling molecules and molecular chaperones and is involved in programmed cell death pathways. The BAG genes in this study have been isolated from the model plant, *Arabidopsis thaliana* (AtBAG4) and the resurrection grass *Tripogon loliiformis* (TlBAG). The NAS genes were isolated from chickpea (CaNas2) and rice (OsNas2) where they catalyze the biosynthesis of nicotianamine (NA) and are involved in Fe uptake and translocation in plants. The ferritin gene was isolated from soybean (GmFerritin) and is an iron storage protein that allows for safe sequestration of iron in a soluble and bioavailable form.

### Plant Material and Seed Sterilization

**FIGURE 3:**
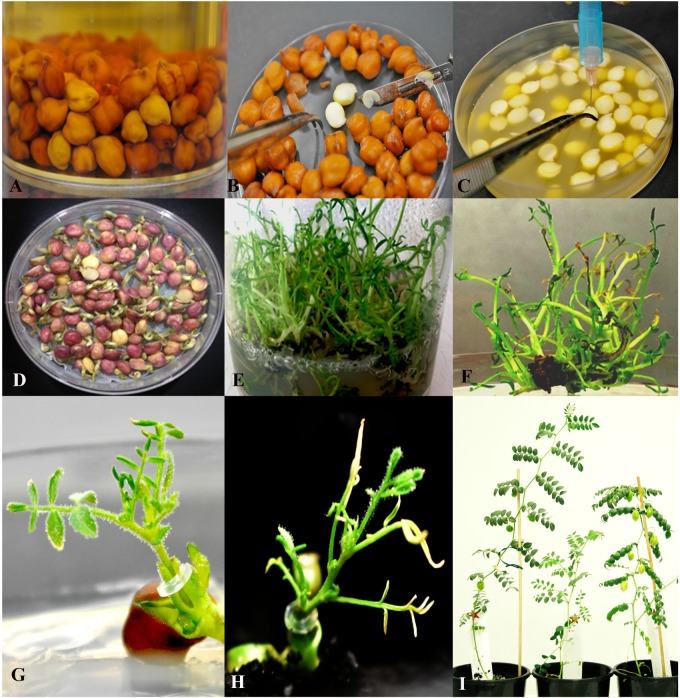
The experimental process of *Agrobacterium*-mediated transformation using half embryo explants. **(A)** Seed sterilization and imbibition in sterile water; **(B)** Dissection of embryos into half along its axis; **(C)** Needle micro-injury; **(D)** 5 day old explants co-cultivated in B5 medium turns purple and start germinating; **(E)** Shoot multiplication in regeneration and selection (RS2) medium; **(F)** Putative shoots selected for *GUS* expression and PCR analysis; **(G)** Grafting of PCR positive *in vitro* shoots; **(H)** Acclimatized grafted shoot in the soil.; **(I)** Fully acclimatized T_0_ plants.

The chickpea cv HatTrick was used for transformation because it is a widely cultivated modern variety in Australia. Approximately 200g of chickpea seeds was rinsed with sterile MilliQ (SMQ) water in 500 mL tissue culture jar followed by disinfecting in 70% (v/v) ethanol for 2 min shaking by hand. The ethanol was replaced with a freshly prepared 1.5% (v/v) sodium hypochlorite solution (diluted with SMQ) and agitated for 7–8 min by hand. Following sterilization, the sodium hypochlorite was decanted, and the seeds were washed 5–7 times with SMQ water. Damaged and discolored seeds were removed, and the remaining seeds were imbibed overnight in SMQ water at ambient room temperature and light conditions (Figure 3A).

### Preparation of *Agrobacterium* Cultures

A starter *Agrobacterium* culture was prepared by inoculating approximately 5 mL of Luria-Bertani (LB) medium (Supplementary Table 1) containing 25 mg/L rifampicin and 50 mg/L kanamycin with 100 μL of AGL1 *Agrobacterium* glycerol stock harboring the gene(s) of interest. This culture was shaken (200 rpm) overnight at 28°C. To prepare the final working culture, 2.5 mL of the starter culture was used to inoculate 500 mL of LB media containing 50 mg/L kanamycin. The final culture was shaken (200 rpm) overnight at 28°C until an absorbance (at 600 nm) of 1.0–1.2 was reached. One hour prior to transformation, 0.5 mL acetosyringone (100 mM) was added to the culture.

### Micro-Injury of Half Embryos and Transformation

Approximately 1 mm was trimmed off the ‘beak’ of the overnight-imbibed chickpea seeds (Figure 3C). The seed coats were removed, and the resulting embryos were bisected along the longitudinal axis with a scalpel to yield two explants each containing half the axis attached to one cotyledon (Figure 3B,C). These explants were used in different co-cultivation conditions (Figure 1). Explants were either uninjured or injured (micro-injury was delivered with a needle five times along the axis as shown in Figure 3C and inset) and incubated in *Agrobacterium* culture at 22°C under 70 μmol s^−1^ m^−2^ of red LED (Valoya L30 spectrum AP67 Conviron Finland) or fluorescent (OSRAM L18W/77 G13 Fluora Germany) lights for 1 h. Four sets of experiments were performed as outlined in Figure 1. To minimize experimental error due to unequally cut half embryos (Supplementary Figure 1B) only regenerable half-embryo explants obtained after 5 days of co-cultivation were counted as the initial explants (250 explants/experiment, repeated thrice for statistical calculations) (Supplementary Figure 1A).

### Co-cultivation of Explants

After 1 h incubation, the bacterial cultures were drained, and the explants were dried on sterile Whatman filter paper. Explants were placed, cut-side-down, on filter paper over Gamborg’s B5 (Gamborg et al., 1976) medium supplemented with 1 mg/L 6-benzylaminopurine (BAP) and 1 mg/L naphthalene acetic acid (NAA) along with 1 ml of acetosyringone (100 mM) (Supplementary Table 1) in a Petri plate and co-cultivated for 5 days at 22 ± 1°C with 70 μmol s^−1^ m^−2^ LED or fluorescent light in a growth room with a 16 h light/ 8 h dark cycle.

### Regeneration and Selection of Shoots

After co-cultivation the explants were transferred to regeneration and selection (RS1) basal MS (Murashige and Skoog, 1962) medium supplemented with 3% sucrose, 10 mM MES,) for shoot 0.5 mg/L BAP, 0.5 mg/L kinetin, 0.05 mg/L NAA, 100 mg/L kanamycin, 25 mg/L meropenem (a member of the family of carbapenems which inhibits the growth of Agrobacterium by interfering with the synthesis of the bacterial cell wall) (Ranbaxy Australia Pty Ltd induction (Supplementary Table 1). The explants were incubated under LED or fluorescent light at 22 ± 1°C, and a 16 h light/ 8 h dark cycle for 19–20 days. Roots and any dead or dying tissues were removed and the surviving shoot clumps were sub-cultured onto fresh regeneration and selection (RS2) medium supplemented with 0.5 mg/L BAP, 0.5 mg/L Kinetin, 100 mg/L kanamycin and 25 mg/L meropenem (Supplementary Table 1). The shoot clumps were sub cultured for one more round for 19–20 days on RS2. At the completion of a third round of selection, clumps of secondary shoots (Figure 3E) that emerged from the base of the explants were ready for PCR testing and grafting. Small shoots from the PCR-positive clump that was not large enough for grafting were further elongated by subculturing on RS3 medium (Figure 3F and Supplementary Table 1).

### GUS Screening of Half Embryos, *in vitro* Shoots and Homozygous Progeny

Histochemical GUS staining was performed according to Jefferson (Jefferson et al., 1987) on 5-day old explants immediately after co-cultivation. Similarly, putative transgenic shoots at the third round of regeneration and selection were stained for GUS expression. The shoots that stained positively for GUS across all tissues were considered to be non-chimeric. In contrast, shoots with blue and non-blue sections were considered to be chimeric. GUS expression in vegetative parts of 2-week-old seedlings of the T_2_ progeny of stable transformants was assayed by dipping seedlings in GUS stain for 24 h at 37°C. Non-transgenic seedlings were stained as controls. Chlorophyll was removed from stained tissues by incubation in 70% ethanol for 24 h.

### PCR Screening of *in vitro* Shoots for Early Detection of Transgenic Events

A single *in vitro* shoot from putative transgenic clumps was harvested and DNA was isolated as described (Thomson and Henry, 1995). PCR was done using GoTaq (Promega) and GUS (*uidA*) gene-specific primers (Fw TGAACATGGC ATCGTGGTGA and Rv GCTAACGTAT CCACGCCGTA). The resulting products were separated via electrophoresis in a 0.8% agarose gel and made visible by staining with SYBR^®^ Safe (Life Technologies).

### Grafting of *in vitro* Shoots

*In vitro* shoots from putative transgenic shoot clumps obtained after the third round of selection were grafted onto non-transgenic rootstocks under *in vitro* conditions as described earlier (Sarmah et al., 2004) except that the rootstock culture medium was replaced with ½ MS without sucrose. The grafted plantlets were incubated at 22 ± 1°C, with 16 h light/8 h dark cycle, for 2 weeks with frequent removal of side shoots until the graft had healed.

### Acclimatization of Grafted Plantlets

Grafted plantlets were removed from the medium and the roots were washed to remove all attached agar. The seed coat was removed to reduce the risk of fungal contamination. The plantlets were transferred to 150 mm diameter pots half-filled with autoclaved mixture (potting mix: perlite, 3:1) and the roots were covered with additional autoclaved mixture. The mixture was gently compressed around the roots and the plantlets were irrigated with 25 mL of tap water. During the transfer, care was taken to keep the plantlets upright and that the graft was above the mixture. The plantlets were covered with a transparent sterilized 500 mL plastic jar and placed in a growth cabinet (CONVIRON model Adaptis A1000 Finland) set at 22 ± 1°C, with 90 μmol s^−1^ m^−2^ LED light for 16 h light/8 h dark cycle. The transparent plastic jar was wiped free of condensation daily and the pots were watered every 3 days. After 10 days or when the plants were nearly touching the walls of the jar, the jar was left partially open for 8–9 days for further acclimation, and then completely removed. The plants were transplanted to 400 mm × 250 mm pot for completion of the life cycle.

### Collection of Transgenic Seed

Seeds (T_1_ generation) were harvested from T_0_ plants and sown in a commercial potting mix (Searles Australia) in 100 mm tube stocks inside a growth cabinet with 90 μmol s^−1^ m^−2^ LED light for 16 h light/8 h dark cycles. The potting mix was kept moist, but not saturated. After emergence, leaf samples were collected from fully expanded leaves for PCR screening. PCR-positive progeny was transplanted to 400 mm × 250 mm pots containing potting mix supplemented with a slow-release fertilizer (Osmocote, Scotts, Australia) for further growth and seed production. Seeds were collected from dried pods and allowed to dry further at room temperature for at least 3 days before being stored at 4°C. The process of harvesting, screening and transplanting was repeated to the 2nd generation, by which stage, homozygous lines had been selected for seed bulking. The time taken from sowing to harvest was 60–63 days.

### Southern Analysis of Transgenic Lines

Early PCR screening using gene-specific primers (Supplementary Table 2) for detecting positive shoots of *AtBAG*, *TlBAG*, *CaNas2*, and *OsNas2* was used to validate transgenic events (data not presented). Southern blot analysis was performed to demonstrate transgene integration and to estimate gene copy number (Tan, 2018). Two single digestions, *Eco*RI and *Nco*I, were used to test the copy number of *AtBAG4* and *TlBAG* lines. *Bam*HI digestion was used for testing the integrity of the *AtBAG4* insertion and *Stu*I/*Nco*I double digestion was used for testing *TlBAG* insertion. *Kpn*I digestion was used to estimate copy number of biofortification genes.

### Statistical Analysis

All data were subjected to one-way ANOVA (MINITAB 17 Statistical Software, 2010). Differences among means for treatments were evaluated by Fischer LSD test at 0.05 probability levels.

## Results

### LED Light Plus Micro-Injury Treatment Enhanced Shoot Regeneration and Transformation Efficiency in Chickpea

Transformed half embryo explants (THEE) grown under LED light (70 μmol s^−1^ m^−2^ light intensity) accumulated anthocyanins in the cotyledons (dark purple) which accompanied the development of healthy green shoot primordia (34.8 ± 1.4%) during the 5 days of co-cultivation (Supplementary Figures 2A,B). Meanwhile, cotyledons of THEE grown under fluorescent light (70 μmol s^−1^ m^−2^ light intensity) accumulated less anthocyanin (cream colored) and developed fewer shoot primordia (31.3 ± 1.6%) (Table 1 and Supplementary Figures 2C,D).

**Table 1:** Transformation frequency and chimerism of chickpea co-cultivated with *Agrobacterium tumefaciens* strain AGL1 harboring pOpt-EBX 35S::*uidA* 35S::*NPT II* binary vector under different treatments and growth conditions.

Treatment	Mean shoot clumps (3rd selection)	Shoot regeneration frequency ± SE %	Mean PCR positive Shoots	Mean Chimeric shoots	Transformation frequency ± SE %	Mean Chimerism±SE %
Micro-injury+ LED	98 ± 3.5^a^	39.2 ± 1.4^a^	5.3 ± 0.72^a^	0 ± 0.0^c^	2.1 ± 0.35^a^	0 ± 0.0^c^
Non-injury + LED	87.0 ± 3.05^b^	34.8 ± 1.2^b^	3.6 ± 0.66^ab^	1 ± 0.0^b^	1.4 ± 0.26^ab^	28.8 ± 4.4^b^
Micro-injury + Fluorescent	39.3 ± 12.0^d^	15.7 ± 4.8^d^	2.6 ± 0.54^bc^	2.3 ± 0.27^a^	1.0 ± 0.26^bc^	91.6 ± 8.3^a^
Non-injury + Fluorescent	78.3 ± 3.3^c^	31.3 ± 1.6^c^	1.6 ± 0.27^d^	1.6 ± 0.27^ab^	0.66 ± 0.13^c^	100 ± 0.0^a^

*Total number of explants (Replicates)/experiment = 250. The values are the means of three experiments (n = 3). The mean values followed by the same letter are not significantly different.*

We observed that half embryo chickpea explants with micro-injury, grown under fluorescent light (70 μmol s^−1^ m^−2^ light intensity) produced fewer (15.7 ± 4.8%) shoots than the non-micro-injured (31.3 ± 1.6%) (Table 1). However, micro-injured THEE grown under LED light generated 2.5-fold more shoots than those grown under fluorescent light (39.2 ± 1.4% and 15.7 ± 4.8% respectively) (Table 1). These results indicate that micro-injury negatively affected shoot regeneration of THEE grown under fluorescent light but not under LED light.

To investigate whether micro-injury also played a role in chickpea transformation efficiency, *in vitro* shoots were screened by PCR at the end of the third round of selection. Micro-injured THEE grown under LED lights produced the highest number of transgenic shoots (5.3 ± 0.72) compared to other treatments (Table 1 and Figure 3E,F). Non-micro-injured THEE grown under LED light produced slightly more transgenic shoots (3.6 ± 0.66) than micro-injured THEE grown under fluorescent light (2.6 ± 0.54) (Table 1) although the difference was not statistically significant. When comparing the mean of PCR positive shoots obtained from micro-injured THEE grown under fluorescent light and that of non-micro-injured THEE grown under the same condition (fluorescent light), a significantly higher mean of PCR positive shoots was recorded for the micro-injured THEE. These results suggest that (i) micro-injury enhances transformation efficiency of chickpea and (ii) the efficiency of chickpea transformation can be further improved by the combined effects of LED lights and micro-injury.

### Chimerism in Chickpea Transformation Was Eliminated Using LED Light Plus Micro-Injury of Explants

**FIGURE 4:**
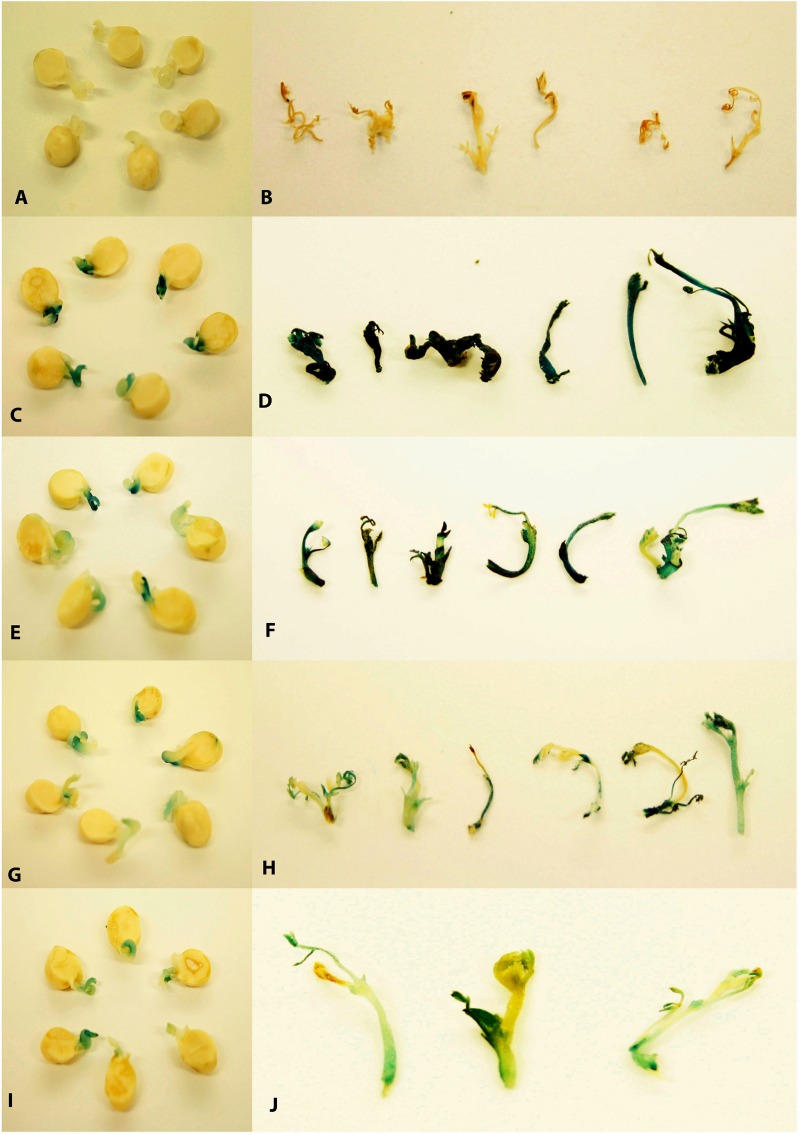
Transient histochemical GUS staining of 5-day old half embryo explants co-cultivated in B5 medium and stable GUS staining of *in vitro* shoots derived after third round of regeneration and selection. **(A,B)** Non-transgenic control without transformation; **(C)** 5-day old half embryo explant with micro-injury under LED light; **(D)** *In vitro* shoots derived after third round of regeneration and selection with micro-injury under LED light; **(E)** 5-day old half embryo explant without micro-injury under LED light; **(F)** *In vitro* shoots derived after third round of regeneration and selection without micro-injury under LED light; **(G)** 5-day old half embryo explants with micro-injury under fluorescent light; **(H)** *In vitro* shoots derived after third round of regeneration and selection with micro-injury under fluorescent light; **(I)** 5-day old half embryo explants without micro-injury under fluorescent light; **(J)** *In vitro* shoots derived after third round of regeneration and selection without micro-injury under fluorescent light.

Chimerism in legume transformation is fairly common and a prime factor in the non-transmission of genes to subsequent generations. Minimizing chimerism in transgenic plants is, therefore, an important target to obtain transmission of transgenes to the T_1_ generation. We investigated whether the combination of LED light and micro-injury can minimize chimerism in THEE. Transient expression of GUS in micro-injured THEE incubated under LED light after 5 days co-cultivation showed intense blue coloration of explants (Figure 4C) as compared to the faint blue color observed in non-micro-injured THEE (Figure 4E). The intensities of blue color on THEE were still lower in micro-injured as well as in non-injured THEE co-cultivated under fluorescent lights (Figure 4G, I). The non-transformed explants (control) grown under both LED and fluorescent light did not develop any blue color (Figure 4A). GUS staining of healthy *in vitro* shoots randomly picked from each set of experiments after the third round of regeneration and selection showed that the entire shoots from micro-injured THEE grown under LED light were intensely blue (Figure 4D). No signs of chimerism were observed (Figure 4D and Supplementary Figure 3C) whereas, shoots obtained from non-micro-injured THEE grown under LED light developed only patches of blue (Figure 4F). Almost all the shoots from fluorescent light-grown THEE with or without micro-injury were chimeric (Figure 4H,J, Supplementary Figure 3B, and Supplementary Table 1). The control shoots grown under LED and fluorescent light were not blue (Supplementary Figure 3A).

#### A Combination of Early Detection by PCR and Grafting Enabled Rapid Production of Transgenic Chickpeas

**FIGURE 5:**
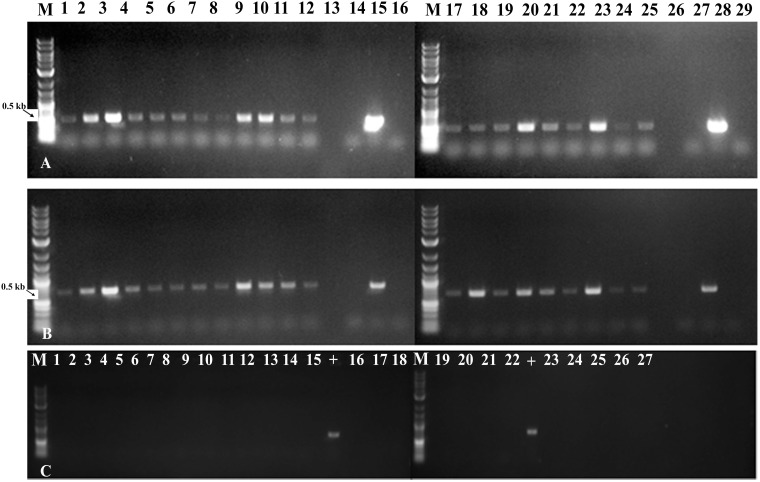
Molecular characterization of fully transformed and chimeric *in vitro* shoots through PCR analysis. **(A)** Amplification of *GUS* (*uidA*) gene from leaves of *in vitro* shoots obtained with micro-injury under LED light (lane 1–6), without injury under LED light (7–12), with micro-injury under fluorescent light (17–22) and without injury under fluorescent light (23–25); **(B)** Amplification of *NPTII* gene from leaves of *in vitro* shoots obtained with micro-injury under LED light (lane 1–6), without injury under LED light (7–12), with micro-injury under fluorescent light (17–22) and without injury under fluorescent light (23–25). Lane 13,26 - Blank; Lane 14,27 - Negative (water) control; Lane 15,28 –Plasmid (*uidA*) control; 16,29 - Non-transgenic control; **(C)** Non amplification of *virC* gene from *in vitro* leaves tissues confirms absence of *Agrobacterium* traces on leaves. Lane – 1–15, 19, 20, 21, 22, 26, 27 samples; 16, 23 – Negative (water) control; 17, 24 - Non-transgenic control; 18, 25 – blank; “+” – *virC* control from *Agrobacterium.*

Using gene-specific PCR primers detected *GUS* (*uidA*) and *npt-II* sequences in genomic DNA of putatively transgenic chickpea at the end of the third round of regeneration and selection (Figure 5A,B, products of 507 bp and 675 bp, respectively). PCR with *virC-* specific primers (Figure 5C) showed that no *Agrobacterium* residue was present.

Approximately 80% of PCR-positive seedlings survived after grafting onto 4-day old non-transgenic rootstocks (data not shown). Grafted shoots were ready for acclimatization after 14 days (Figure 3G). Thus, early screening at the third round of selection enabled rapid screening of positive plants that were grafted to speed up generation of transgenic plants.

### Acclimatization and Accelerated Production of Subsequent Generations

**FIGURE 6:**
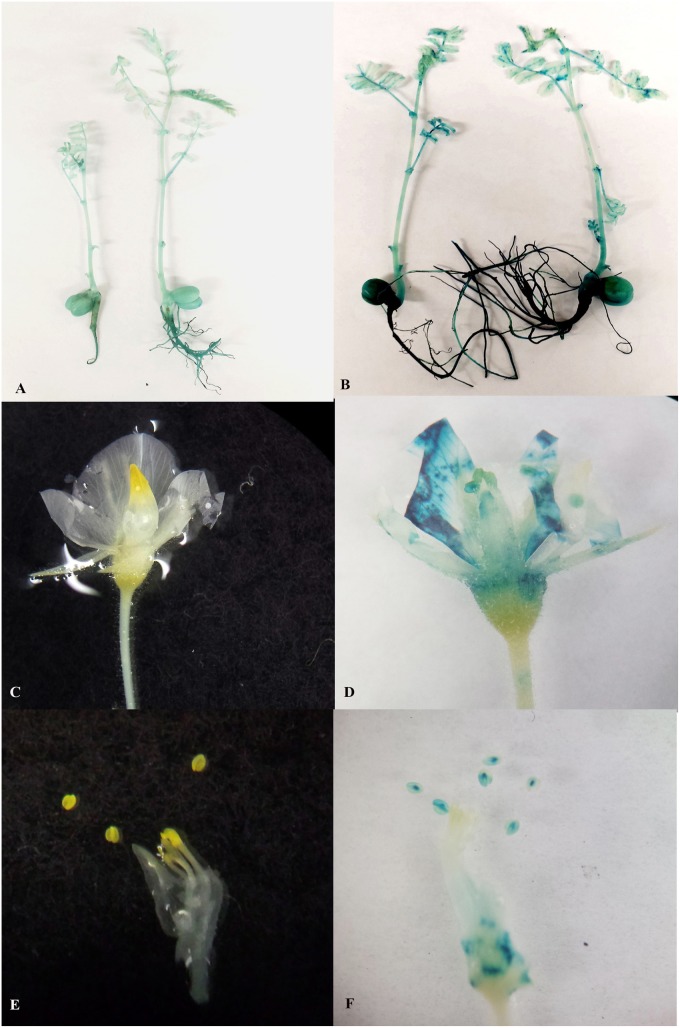
Stable GUS expression in vegetative and reproductive parts of 2nd generation progeny. **(A)** Two week old non transgenic seedlings without any GUS expression; **(B)** Two week old transgenic seedlings with GUS expression in shoots roots, leaves and cotyledons; **(C)** Flowers of non-transgenic seedlings without any GUS expression; **(D)** Flowers of transgenic seedlings with GUS expression in sepals, petals stamens and stigma; **(E)** Stamens of non-transgenic seedlings without any GUS expression; **(F)** Stamens of transgenic seedlings with GUS expression.

The overall efficiency of any transformation protocol depends on the number of plantlets that survive acclimatization and in our initial attempts we found that chickpea acclimatization was difficult. This appeared to be mainly due to the fact that chickpea is very sensitive to excess moisture. When maintained under high humidity, grafted chickpea leaves and shoots became extremely susceptible to fungal infection. We found that perlite in the autoclaved mixture (potting mix: perlite, 3:1) that slowly released water to the plantlet prevented infection. It was also important to pre-sterilize soil mixtures and pots and to remove moisture regularly from the pot covers. In summary, about 70% of the plantlets could be successfully acclimatized in 18–19 days (Figure 3H,I; data not shown). As soon as the transgenic chickpea plantlets were acclimatized they could be transferred to bigger pots and grown under high-intensity LED to produce 10–12 mature T_1_ generation seeds within about 9 weeks. Using this improved protocol, a transgenic event can be taken to the 2nd generation within 8 months after transformation (Figure 1). By this stage, homozygous lines had been selected. It was essential to examine whether the T_0_ transgenic events generated using LED light plus micro-injury treatment transmitted the transgene stably in subsequent generations. All the vegetative parts such as leaves, shoots and roots and cotyledons of T_2_ progeny turned blue. The reproductive tissues such as corolla, petals, stamen and pistil were also blue (Figure 6D,F). The Vegetative and reproductive parts of non-transgenic control plants remained white (Figure 6A–D).

### Generation of Low Copy Number Stable Transgenic Lines With Different Gene Cassettes

**FIGURE 7:**
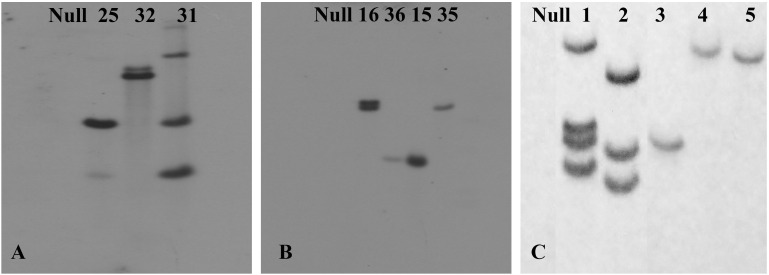
Southern blot hybridization of the transgenic plants with **(A)** a probe for the gene *AtBAG4* to detect the presence of integrated transgene; **(B)** a probe for the gene *TlBAG* to detect the presence of integrated transgene; **(C)** a probe for the selection marker, *NPTII*, which was linked to and assumed to act as a surrogate for the presence of the OsNas2 and *CaNas2* transgenes.

To assess the transformation efficiency quantitatively, we monitored the entire process during regeneration of *in vitro* shoots under selection using the *uidA* gene and observed a 2.1 ± 0.35% transformation efficiency (Figure 7). The improved protocol was used to produce transgenic chickpea lines with *TlBAG* and *AtBAG4* genes as well as *OsNas2* and *CaNas2* genes. Southern blot analysis of T_1_ plants showed that two out of three *AtBAG4* lines (#25 and #32) contained a single copy of the gene. For *TlBAG* lines (#15, #16, #35 and #36) three out of four lines had single copies (Figure 7A,B). Similarly, three of the five *CaNas2* lines (#3, #4, and #5) had single copies of the *NPTII* gene (Figure 7C). Segregation of the transgene in the first generation (T_1_) progeny of *TlBAG* (TP-35, TP-36), *CaNas2* (TP-5), as well as the two copies contained in *AtBAG4* (TP-32), was determined by qPCR (Supplementary Table 2). Thus, micro-injured explants and growth under LED not only produced stable GUS transgenics but also produced chickpeas with a range of other genes that were inherited in subsequent generations (Table 2).

**Table 2:** Segregation of transgenes in T_1_ progeny of chickpea plants transformed with different genes.

T_0_ lines	Gene	Copy no	Total no of T_1_ seeds	PCR (+) progenies	Plants (−) progenies	Observed ratio	χ^2^ value^a^	*P*-value
TP-35	*TlBAG*	1	6	4	2	2:1	0.22	0.639
TP-36	*TlBAG*	1	4	3	1	3:1	0	1
TP-32	*AtBAG4*	2	58	37	21	1.7:1	3.8	0.051
TP-5	*CaNas2*	1	19	12	7	1.6:1	1.42	0.23

*^a^Against 3:1 (critical χ^2^_0.05;1_ value = 3.84).*

## Discussion

Genetic improvement in chickpeas through GE and genome editing will require a robust genetic transformation system that can efficiently generate numerous transgenic events. Initial explants used for any transformation is of outmost importance and a previous report of chickpea transformation (Sarmah et al., 2004) indicated that half embryo explants were the most suitable. These explants have terminal or axillary meristems that serve as sources of totipotent cells (Swathi Anuradha et al., 2008). Therefore, we examined various parameters that could improve the transformation efficiency as well as eliminate chimerism using these meristematic embryo explants.

Of the various parameters, light is vital for regulating growth and development during plant tissue culture (PTC) (Reuveni and Evenor, 2007; Godo et al., 2011). Therefore, the effects of LED and fluorescent light on chickpea regeneration were tested. In this study, LED light Valoya AP67 provided 53% of red, 17% far-red, 16% green and 14% blue spectrum with a total of 83% in the photosynthetically active region (PAR) between 400 and 700 nm. The ratios of red to far red (R: FR) and blue to green (B:G) determines plant growth (Darko et al., 2014; Naznin et al., 2016; Ouzounis et al., 2016). The spectrum of AP67 is enriched in red. Red light is the most effective spectrum harvested by photoreceptor phytochrome (phy), and gets transferred to chlorophyll molecules to perform photosynthesis and produce sugars and carbons (Darko et al., 2014). PhyB is a protein photoreceptor with a covalently attached light sensitive chromophore, whose activity is mainly controlled by the R:FR ratio. A high R:FR ratio activates phyB which triggers the proteasome-mediated destruction of the transcription factor, PIL5 (Oh et al., 2004, 2006). Under low PIL5, transcription of gibberellic acid (GA) inhibitors viz. gibberellic acid insensitive (GAI) and repressor of GA1 (RGA), is lowered and GA synthesis occurs which helps promote seed germination (Oh et al., 2007). A low R:FR ratio stimulates abscisic acid (ABA) synthesis which prevents seed germination (Piskurewicz et al., 2009). Phytohormones ABA and GA antagonistically regulate various developmental processes and responses to environmental cues, such as light, temperature, and abiotic stresses (Weiss and Ori, 2007; Vanstraelen and Benková, 2012; Golldack et al., 2013). Both these phytohormones are present in developing chickpea seeds (Slater et al., 2013) and mature dry seeds are reported to contain high levels of ABA, which decreases after germination (Iglesias and Babiano, 1997). Endogenous ABA levels increase 3-to 5-fold upon wounding in several plant species (Peña-Cortés et al., 1995). This increase is not restricted to the damaged tissue but can occur systemically (Pena-Cortes et al., 1991). From these studies, we speculate that dissection and micro-injury of half embryos may lead to an increase in ABA levels leading to reduced emergence of shoot primordia under fluorescent light. The red component of LED light is expected to increase GA levels and promote initiation and growth of shoot primordia. Furthermore, there are several reports that LEDs stimulate organogenesis as well as embryogenic responses in other plants (Gupta and Jatothu, 2013; Gupta and Agarwal, 2017). AP67 LED also has a moderate amount of blue and green light known to increase photosynthesis leading to enhanced leaf and plant size in other plants (Darko et al., 2014; Naznin et al., 2016; Ouzounis et al., 2016).

Endogenous ABA increases under stress (Yoshikawa et al., 2007; Kondo et al., 2012) and acts as a signal transduction substance to induce secondary metabolite substances to protect against stresses (Cutler et al., 2010). In the current study, dissection and micro-injury of half embryo explants followed by transformation with hyper aggressive *Agrobacterium* (AGL1) endures sufficient stress to explants which presumably increase endogenous ABA that results to increase reactive oxygen species (ROS) reported to cause oxidative stress when accumulated at a high level in plant cells (Xu and Rothstein, 2018). Anthocyanin acts as an ROS scavenger and its accumulation has been important in contributing protection against ROS (Nakabayashi et al., 2014; Zhang et al., 2015). LED-mediated increase in the accumulation of primary metabolites such as anthocyanins, polyphenols and flavonoids (Heo et al., 2012; Lee et al., 2016) that promotes enhanced growth and development of plants has been reported (Misyura et al., 2013). AP67 LED lights have been reported to enhance anthocyanin in pomegranate seedlings (Bantis et al., 2018). Formation of red color due to anthocyanin pigments on cotyledons of THEE of chickpea grown under AP67 LED lights was comparatively much higher than the fluorescent counterpart. Thus, might have provided protective role for increased survivability of dissected micro-injured explants leading to enhanced recovery of transformed shoots. The red LEDs have a pronounced effect on anthocyanin accumulation due to the increased expression of the anthocyanin biosynthesis genes, *MdMYB10* and *MdUFGT* (Lekkham et al., 2016). Consistent with this, transformation frequency was increased up to 10 and 20% in transgenic strawberry and apple that overexpressed the transcription factor *MYB10* which leads to anthocyanin production (Kortstee et al., 2011).

Most of the previous reports on chickpea transformation reports chimerism and the protocols were largely non-repeatable across different laboratories. Since Sarmah et al., 2004 reported only 0.8% transformation efficiency in chickpea (Semsen cv) which was also repeatable by Acharjee et al., 2010 in chickpea (ICCV 89314 cv), we first investigated the transformation efficiency in chickpea (HatTrick cv) using this repeatable protocol and then further increased the transformation efficiency by modifying various parameters. In this investigation, it was observed that performing the transformation with chickpea (HatTrick cv) by following Sarmah et al., 2004 protocol (without micro-injury under fluorescent light) a transformation efficiency of 0.66% was obtained. However, on modifying the key steps, it was possible to increase the transformation efficiency to about 3.6-fold. Thus, the increase in transformation efficiency is solely due to improvements in key steps of existing protocol and is not due to genotypic variations. This modified protocol can therefore be easily adopted for improving other chickpea varieties. There are numerous reports of improved transformation efficiency using micro-injury on explants in recalcitrant species. In previous studies, it was suggested that micro-injury activates stress response pathways and improves transformation efficiency in legumes (Saini et al., 2007; Bakshi et al., 2011). We found that micro-injuring the half-embryo explants using a fine needle negatively affected shoot regeneration frequency of THEE grown under fluorescent light but not under LED light. Here we show that micro-injury alone was not sufficient for maximizing the number of PCR positive shoots as even non-injured explants grown under LED light formed several PCR positive shoots. However, when micro-injury was combined with LED growth there was a significant improvement in transformation efficiency. Injuring plant tissue before co-cultivation has also been shown to significantly enhance transformation efficiency in *Vitis vinifera* (Dutt et al., 2007), *Eucalyptus tereticornis* (Aggarwal et al., 2011) and highly recalcitrant legumes like mungbean and cowpea (Bakshi et al., 2011). We speculate that the enhanced transformation efficiency with micro-injury may be due to the fact that wounding of tissue helps bacterial penetration deep into the tissue facilitating the accessibility of plant cells to *Agrobacterium* as well as stimulating the induction of *Vir* genes as a result of phenolics secretion (Stachel et al., 1985) leading enhanced bacterial efficiency for T-DNA delivery (Binns and Thomashow, 1988). Although micro-injury plays an important role in efficient T-DNA delivery that leads to enhanced frequency of transformed cells, regeneration of shoots from these micro-injured cells would be possible if favorable conditions mitigate the stress level incurred due to injury and dissection of explants. The possibility of stress reduction through conventional fluorescent light is feeble whereas the same can be achieved through efficient red LED light as explained above. Micro-injuring secretes phenolic compound and addition of acetosyringone for *Vir* gene induction of *Agrobacterium* can actually exceed the threshold level which might be lethal to explants. To minimize the detrimental effects on micro-injured explants, pre-induction of *Agrobacterium* was done initially for 1h followed by final induction during co-cultivation with micro-injured half embryo explants in B5 medium supplemented with acetosyringone and growth regulators. This procedure has also been found to be very effective for higher survivability and better transformation efficiency in recalcitrant cowpea (Bakshi et al., 2011).

Although several workers have reported generation of T_0_ chickpea transgenic events (Kar et al., 1996; Krishnamurthy et al., 2000; Sarmah et al., 2004; Tewari-Singh et al., 2004; Mishra et al., 2013; Tripathi et al., 2013) however; many have not reported stable inheritance of the genes. In a recent reports, out of initial 46 confirmed T_0_ lines, only two transgenic could successfully transmitted the single copy transgene in T_2_ progeny (Chakraborty et al., 2016). Chimerism could be a reason for the unstable inheritance as reported earlier in legumes. The prevalence of chimerism across different species is thought to be due to the multicellular origin of shoot organogenesis (Poethig, 1989; Zhu et al., 2007) that may allow protection of non-transgenic cells by the surrounding transformed cells (Park et al., 1998; Domínguez et al., 2004) or the ineffectiveness of selective agents in certain species having endogenous tolerance (Rakosy-Tican et al., 2007). We observed chimeric events from non-micro-injured explants grown under fluorescent light. The scattered GUS expression on *in vitro* shoots demonstrated that previous protocols employing fluorescent light without injury of explants had a higher probability of generating chimeric shoots. These chimeric events may not necessarily produce positive (T_1_) progeny if seeds are produced from non-transgenic portions of the parental line. Sarmah (Sarmah et al., 2004) reported non-transmission of the transgene in T_1_ progeny in four of transgenic events which was likely due to chimerism. However, by micro-injuring the explants and growth under LED light helped to eliminate chimerism and to produce only fully transformed shoots.

PCR screening of the fully transgenic *in vitro* shoots at the end of third round of selection rapidly generated transgenic chickpea. In contrast, the previous protocol required six rounds of regeneration and selection (Sarmah et al., 2004). We slightly modified the grafting method and used ½ MS agar to enable rapid and healthy growth of the transgenic scion. In previous studies 16 weeks of culture were required to produce transgenic chickpea plants in the glasshouse (Sarmah et al., 2004; Senthil et al., 2004). That has now been reduced to 9 weeks using LED lights. The shortening of generation turnover in legumes such as pea through increased red: far red (R: FR) ratio of light spectrum has been reported earlier (Ribalta et al., 2017). Using this protocol, it is possible to obtain non-chimeric, stable, and homozygous transgenic chickpea in the T_2_ generation within 8 months. The protocol was robust enough to successfully generate stress tolerant chickpea lines with BAG genes isolated from *Arabidopsis* and *Tripogon loliiformis*, respectively. Furthermore the protocol was used to transform chickpea with multiple genes (*Nas2*, *Ferritin* and *NPT-II* placed in the same construct to generate biofortified chickpea (Tan et al., 2018).

## Conclusion

The method described in this work provides a robust and reliable technique to obtain non-chimeric and stable transgenic chickpeas with improved transformation efficiency and transgene inheritance. The technique is simple and was illustrated with a variety of gene constructs aimed at agronomic and nutritional improvement of chickpea.

## Author Contributions

SDB, BW, SM, and TJH designed the experiments. SDB, AC, and HL performed the experiments. SDB and ZT generated the data for the pictures. SDB, MK, TMLH, BW, SM, and TJH drafted the manuscript. All authors read, revised, and approved the final manuscript.

## Conflict of Interest Statement

The authors declare that the research was conducted in the absence of any commercial or financial relationships that could be construed as a potential conflict of interest.
