# Supplementary material for: Robust Genetic Transformation System to Obtain Non-chimeric Transgenic Chickpea
Source: Front Plant Sci. 2019 Apr 26;10:524. doi: 10.3389/fpls.2019.00524 (PMC6498970; doi:10.3389/fpls.2019.00524)
Supplement: Supplementary file 1 [file Data_Sheet_1.pdf]

Supplementary Material

Robust Genetic Transformation System to Obtain Non-Chimeric Transgenic Chickpea

Das Bhowmik, S.S[1], Cheng, A. [1], Long, H[1], Tan Z.H.G[1], Hoang, T.M.L[1], Karbaschi, M.R.[1], Williams, B.[1], Higgins T.J.V.[1,2], Mundree, S.G.[1]*

*** Correspondence:**
Prof Sagadevan Mundree
sagadevan.mundree@qut.edu.au

1 Supplementary Figures and Tables

1.1 Supplementary tables

Supplementary Table 1. Chemical composition of different media used for regeneration and transformation of chickpea

Medium (1L)	Composition	Growth Regulators	pH
Luria-Bertani (LB) liquid	1% (w/v) Bacto-tryptone, 0.5% (w/v) Bacto-yeast extract, 170mM sodium chloride	-	7.0
Gamborg B5	100mL B5 Macro Stock (10x), 1mL B5 Micro Stock (1000x), 10mL Fe-EDTA Stock (100x), 10mL B5 Vitamins Stock (100x), 30g sucrose, 1.95g MES monohydrate, 1mL BAP (0.5mg/L), 1mL NAA (0.5mg/L), 8g Difco agar, 1mL Acetosyringone (100mM)	1 mg/L BAP and 1 mg/L NAA	5.8
Regeneration and selection (RS1)	4.43g Murashige & Skoog (MS) powder, 30g sucrose, 1.95g MES monohydrate, 8g Difco agar	0.5 mg/L BAP, 0.5 mg/L Kinetin, 0.05 mg/L NAA, 100 mg/L Kanamycin and 25 mg/L Meropenem	5.8
Regeneration and selection (RS2)	4.43g MS powder, 30g sucrose, 1.95g MES monohydrate, 8g Difco agar	0.5 mg/L BAP, 0.5 mg/L Kinetin, 100 mg/L Kanamycin and 25 mg/L Meropenem	5.8
Regeneration and selection (RS3)	4.43g MS powder, 30g sucrose, 1.95g MES monohydrate, 8g Difco agar	0.5 mg/L BAP, 0.5 mg/l Kinetin, 50 mg/L Kanamycin and 25 mg/L Meropenem	
Grafting medium	2.2g MS powder, 1.95g MES monohydrate, 8g Difco agar	-	5.8

Supplementary Table 2. Primer sequences used in PCR screening of transgenic chickpea plants

Gene	Forward Primer	Reverse Primer	Expected Amplicon (bp)
AtBAG4	TGGTGGTATGCTCGTCCAAC	GAGGGTTCAAGCTTCCGACA	710
TlBAG	GAAGAGGGTCATTGCCCAGG	CGTCTCCCACTGCGTTGTTA	514
uidA	TGAACATGGCATCGTGGTGA	GCTAACGTATCCACGCCGTA	507
CaNas2	GCATGTCACCAATCCCCAAC	CGCAGCATCAAAAGTGCTCC	568
OsNas2	CTGAGCAAGCTGGAGTACGA	TCAGACGGATAGCCTCTTGG	660
NPTII	ATTCGGCTATGACTGGGCAC	TAAAGCACGAGGAAGCGGTC	657

1.2 Supplementary Figures

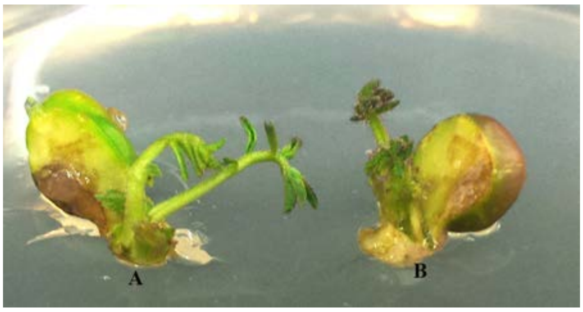

Supplementary Figure 1. (A) Regeneration of shoot primordia from equally dissected half-embryos; **(B)** Unequally dissected half-embryos without regenerable shoot primordia

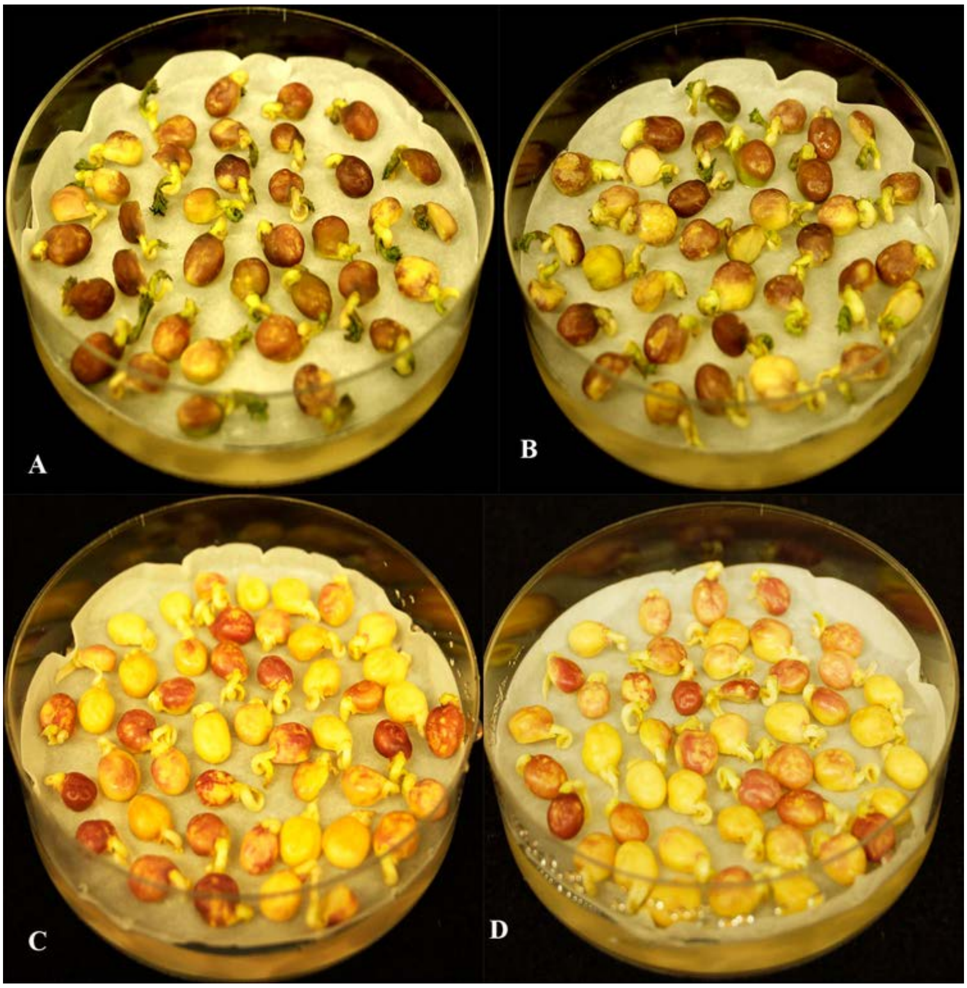

Supplementary Figure 2. Shoot initiation and development of anthocyanin on cotyledons after co-cultivation of half embryo explants for 5 days in B5 medium under different condition **(A)** Transformed half-embryo explants without injury under LED light; **(B)** Transformed half-embryo explants with injury under LED light; **(C)** Transformed half-embryo explants without injury under fluorescent light; **(D)** Transformed half-embryo explants with injury under fluorescent light

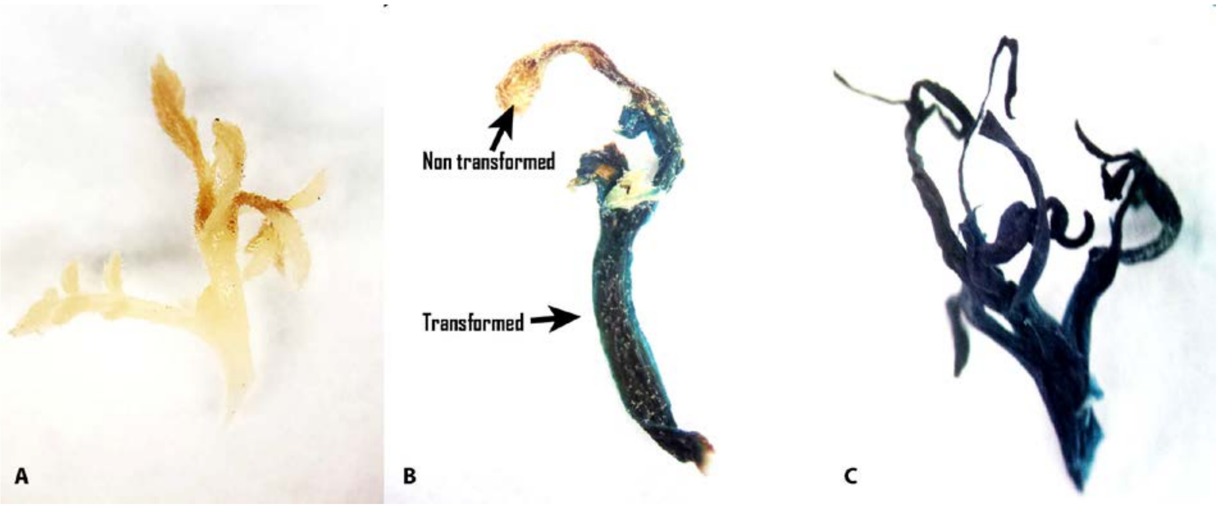

Supplementary Figure 3. Chimeric and fully transformed *in vitro* shoots detected through GUS staining **(A)** Non-transformed *in vitro* shoot stained with GUS solution remained brownish yellow; **(B)** Chimeric shoot with patches of untransformed (brownish yellow) and transformed (blue) portion obtained under fluorescent light without injury; **(C)** Fully transformed shoots obtained from injured explants grown under LED light turned blue in colour after GUS staining.
